# Shared phonological networks in frontal and temporal cortex for language production and comprehension

**DOI:** 10.1093/cercor/bhaf275

**Published:** 2025-10-13

**Authors:** Xenia Dmitrieva, Jean-Luc Anton, Amie Fairs, Bissera Ivanova, Julien Sein, Bruno Nazarian, Sophie Dufour, Friedemann Pulvermüller, Elin Runnqvist, Kristof Strijkers

**Affiliations:** Aix-Marseille Université (AMU) & CNRS, Laboratoire de Parole et Langage (LPL), Aix-en-Provence, France; Aix-Marseille Université (AMU), Institut de Neurosciences de la Timone (INT), Centre IRM-INT, Marseille, France; Aix-Marseille Université (AMU) & CNRS, Laboratoire de Parole et Langage (LPL), Aix-en-Provence, France; Aix-Marseille Université (AMU) & CNRS, Laboratoire de Parole et Langage (LPL), Aix-en-Provence, France; Aix-Marseille Université (AMU), Institute for Langage, Brain and Communication (ILCB), Marseille, France; Aix-Marseille Université (AMU), Institut de Neurosciences de la Timone (INT), Centre IRM-INT, Marseille, France; Aix-Marseille Université (AMU), Institut de Neurosciences de la Timone (INT), Centre IRM-INT, Marseille, France; Aix-Marseille Université (AMU) & CNRS, Laboratoire de Parole et Langage (LPL), Aix-en-Provence, France; Freie Universität Berlin (FUB), Brain Language Laboratory, Department of Philosophy and Humanities, Berlin, Germany; Aix-Marseille Université (AMU) & CNRS, Laboratoire de Parole et Langage (LPL), Aix-en-Provence, France; Aix-Marseille Université (AMU), Institute for Langage, Brain and Communication (ILCB), Marseille, France; Aix-Marseille Université (AMU) & CNRS, Laboratoire de Parole et Langage (LPL), Aix-en-Provence, France; Aix-Marseille Université (AMU), Institute for Langage, Brain and Communication (ILCB), Marseille, France

**Keywords:** FMRI, language comprehension, language production, neurolinguistics, phonology

## Abstract

In this functional magnetic resonance imaging study, we investigated whether language production and understanding recruit similar phoneme-specific networks. We did so by comparing the brain’s response to different phoneme categories in minimal pairs: Bilabial-initial words (eg “monkey”) were contrasted to alveolar-initial words (eg “donkey”) in 37 participants performing both language production and comprehension tasks. Individual-specific region-of-interest analyses showed that the same sensorimotor networks were activated across the language modalities. In motor regions, word production and comprehension elicited the same phoneme-specific topographical activity patterns, with stronger haemodynamic activations for alveolar-initial words in the tongue cortex and stronger activations for bilabial-initial words in the lip cortex. In the posterior and middle superior temporal cortex, production and comprehension likewise resulted in similar activity patterns, with enhanced activations to alveolar- compared to bilabial-initial words. These results disagree with the classical asymmetry between language production and understanding in neurobiological models of language, and instead advocate for a cortical organization where phonology is carried by similar topographical activations in motor cortex and distributed activations in temporal cortex across the language modalities.

## Introduction

The production and comprehension of language must be continuously coordinated in dialog and conversation (Pickering and Garrod 2013; Levinson 2016). Therefore, understanding how production and comprehension interact with each other is important for cognitive and neurobiological models of language (Kerr et al. in press). Nevertheless, historically, the language modalities have been studied separately. This was in part due to observations with aphasic patients, who were once classified in “sensory” and “motor aphasias”, following observations that language modalities can be selectively dissociated by neurological disease (Wernicke 1874; Lichtheim 1885; Geschwind 1979); an observation which seemed to receive further support from neuroimaging work with healthy participants (Price 2012). However, several findings do not support a strict separation of production and comprehension: While “sensory aphasia” is well-known to be characterized not only by comprehension deficits, but by production deficits too (Damasio and Damasio 1980; Caplan 1987), even aphasias once believed to be entirely “motor” come with well-characterized comprehension deficits (Caramazza and Zurif 1976; Miceli and Caramazza 1988). Similarly, fine-grained analyses of language production and comprehension behavior in healthy language users suggests a closer relationship between the two systems than previously assumed (Dell and O'Seaghdha 1992; Levelt et al. 1999; Meyer et al. 2016). However, neurometabolic and neurophysiological imagining studies are necessary for more precisely defining that relationship and, potentially, the degree of overlap between the brain’s production and comprehension networks.

Consequently, in recent years, cross-modal language research has notably increased, and several neuroimaging studies have contrasted production and comprehension for conversational dynamics and sentence processing. This important work demonstrated strong neural overlap across the language modalities for syntax, message-level semantics and story-level content (Stephens et al. 2010; Segaert et al. 2012, Dikker et al. 2014; Silbert et al. 2014; Giglio et al. 2022, 2024; see also Fedorenko et al. 2024; but see Matchin and Wood 2020). We note however, that most of these studies engaged their participants in tasks that exceed basic language comprehension or production per se, and involve a range of complex cognitive processes such as theory-of-mind, indirect inferences of utterance meaning, prediction of upcoming phrasal content, memory recall, attention and so forth. While we certainly agree that all of these processes are necessarily paired with the use and understanding of the basic building blocks of language, nevertheless, when it comes to drawing strong conclusions on any shared brain mechanisms for language production and comprehension, it is imperative to distinguish specific language representations from any of the aforementioned accompanying and higher-level cognitive operations triggered by the complex linguistic tasks. Therefore, here we aim to investigate the degree of neural overlap between the language modalities with functional magnetic resonance imaging (fMRI) using well-known, basic tasks at the word-level, and specifically focus on the question whether the brain signatures for phonological processing are shared between the production and comprehension of language.

Focusing on phoneme representations at the word-level is relevant since current brain language models differ in their assumptions whether phonological processing is asymmetrical between word production and comprehension (Indefrey and Levelt 2004; Hickok and Poeppel 2007; Indefrey 2011; Hickok 2012; de Zubicaray 2023), or whether it is fully integrated across the language modalities (Pulvermüller 1999, 2018; Pulvermüller and Fadiga 2010; Tourville and Guenther 2011; Strijkers and Costa 2016; Kerr et al. 2023). Furthermore, unlike for semantics (Binder et al. 2009) or syntax (Hagoort and Indefrey 2014), there are only two studies we know of that have directly compared phonological processing between the production and comprehension of meaningful words within the same participants. In Okada and Hickok (2006) fMRI data demonstrated neural overlap for word production and comprehension in the posterior superior temporal gyrus and sulcus (pSTG and pSTS), regions which are often linked to phonological processing (Indefrey and Levelt 2004). However, in that study, no specific phonological manipulation was targeted, which makes inferences about phonology indirect; speech production was assessed covertly, which has been criticized to be distinct from natural overt production (Strijkers and Costa 2011); and the number of tested participants was rather low (10 participants). More recently, Fairs et al. (2021) contrasted with EEG the passive perception and overt production of words varying in phonotactic frequency (ie how often phonemes in a word co-occur: Vitevitch and Luce 1999). The authors observed early event-related potential (ERP) modulations elicited by phonotactic frequency in both production and comprehension, making them conclude that the same rapid parallel brain dynamics underpin both modalities. Nevertheless, overlapping temporal dynamics between the production and comprehension of words does not per se mean that also the cortical sources of phonological processing overlap. In sum, the few neurophysiological data available thus far supports the idea of some cross-modal overlap in a word’s phonology, but these data need to be extended in scope, precision, and power. The current study aims at doing so by comparing with fMRI the overt production and passive perception of words within the same participants, and utilizing a specific phonological manipulation, which allows to directly assess whether phoneme-specific activation in the cerebral cortex is overlapping or not across the language modalities.

To elicit such phonology specific brain activity, we compare the mapping of word-initial phonemes in minimal pairs, which are words that differ only in a single sound. Concretely, we contrast minimal pairs with either bilabial- or alveolar-initial word phonemes (eg “/**b**allon/vs./**t**alon/” in French; English translation: ball vs. heel). Using this contrast is interesting because it concerns an unambiguous phonological manipulation which, unlike formal variables such as word length or neighborhood density, is not correlated with other stages of linguistic processing (Strijkers et al. 2010). Also, the manipulation of bilabial and alveolar phonemes has already been successfully applied to both the production (Guenther et al. 2006; Bouchard et al. 2013; Murakami et al. 2015; Strijkers et al. 2017) and perception of speech (Fadiga et al. 2002; Pulvermüller et al. 2006; D’Ausilio et al. 2009; Möttönen and Watkins 2009; Schomers et al. 2015). This makes it an ideal contrast to utilize in a study that aims to directly compare both modalities. Of particular relevance are the studies by Pulvermüller and colleagues in perception (2006) and Strijkers and colleagues in production (2017). In an fMRI study, Pulvermüller et al. found that listening to bilabial and alveolar speech syllables (eg “/ba/vs./ta/”) activated the motor cortex in a topography-specific manner. Bilabial speech sounds activated more strongly those regions responsible for moving our lips and alveolar speech sounds activated more strongly the motor cortex responsible for moving our tongue. Using magnetoencephalography, Strijkers et al. extended this finding to speech production with real words (eg “/Donkey/vs./Monkey/”), observing topography-specific activation of the motor cortex within 200 ms after presentation of to-be-uttered object names. In addition, both studies also reported phoneme-specific activations for the bilabial and alveolar speech sounds in the pSTG, suggesting a correspondence in fronto-temporal networks sensitive to phoneme processing in production and comprehension. However, several studies using electrocorticography (ECoG) did not observe a clear role for place of articulation in the pSTG during speech perception but rather distributed acoustic responses (Mesgarani et al. 2014; Bhaya-Grossman and Chang 2022; Leonard et al. 2024). And similarly for the motor system, one ECoG study reported that while the articulation of speech sounds did result in motor somatotopy, the perception of speech sounds did not, and instead triggered distributed activations sensitive to acoustic features in the motor cortex mimicking the neural responses in the auditory cortex (Cheung et al. 2016; but see Skipper et al. 2017).

**Fig. 1:**
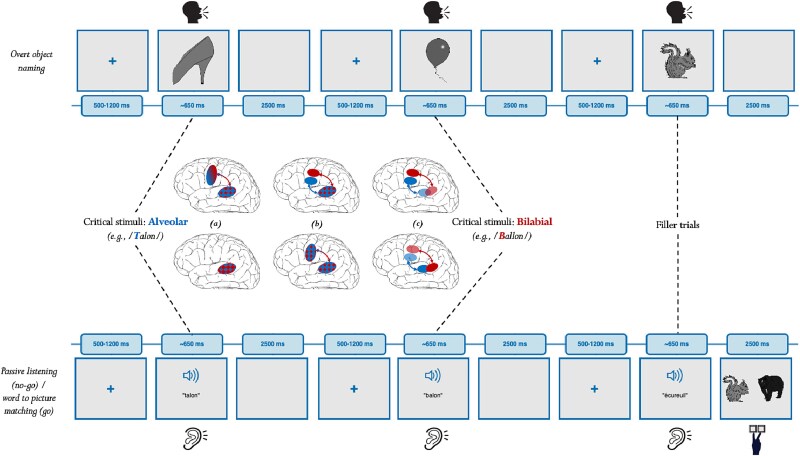
Design and predictions of the experiment. The upper panel depicts the trial structure for the language production task (object naming) and the lower panel for the language comprehension task (passive listening). The same lexical units are targeted in both conditions, naming of object pictures by producing minimal pairs of alveolar-initial words (blue) vs. bilabial-initial words (red) and listening to the same spoken word forms. The middle panel depicts in a simplified and schematic manner the possible activation patterns for this alveolar-bilabial contrast between the language modalities in the motor cortex and STG: a) modality-specific with both STG and motor cortex activation in production, but only STG activation in perception (which furthermore could be different from the STG activation in production). b) Shared, but functionally distinct activation across the modalities with topographical activation in the motor cortex and distributed activation in the STG during production, and distributed activation both in the motor cortex and STG during comprehension. c) Fully integrated activation across the modalities with topographically specific activation patterns in the motor cortex and overlapping activation in the STG (possibly also displaying a somewhat similar topographical response).

In sum, whether production and comprehension rely on overlapping phonological networks and if so, whether such overlap involves both frontal and temporal brain regions, remains an unresolved question. Moreover, and crucial, a direct test that examines whether phonology-sensitive cortical regions activated during production are the same as those activated during comprehension -within the “same brain” and for “meaningful language”—is still lacking. Exploring both modalities within the same brain is important to establish in an individual-specific manner that the same cortical regions are involved in speaking vs. listening, and exploring meaningful language is important since phonological information in arbitrary sounds or syllables may be processed differently from phonemes embedded in structured words (Miceli et al. 1980; Schomers et al. 2015; Strijkers 2016). To address this gap, in the present study we record fMRI in 37 participants who do both a standard production (object naming) and perception task (passive listening) for the same minimal word pairs which only differed in place of articulation (Fig. 1). Furthermore, we relied on a precise regions of interest (ROIs) approach (see the Methods section), which focusses on specific phoneme-sensitive brain regions in the motor cortex and the superior temporal cortex to ensure that any activation we observe in those regions are related to the main manipulation (place of articulation) and cannot be accounted for by confounding factors. With this design we will verify whether bilabial and alveolar phoneme representations of words elicit similar activation patterns across the language modalities in frontal and temporal cortex. Different outcomes are possible (Fig.1): (i) “Modality-specific” activation where the STG is active in both modalities but motor cortex activation is exclusive to production (Hickok and Poeppel 2007; Hickok 2012); furthermore, within temporal cortex there could even be local separation between production and perception “lexemes” (Indefrey and Levelt 2004; Indefrey 2011); (ii) “Shared but functionally distinct” activation, where STG and motor cortex are activated in both modalities, but with different activation profiles: distributed during comprehension and topographically organized during production (Cheung et al. 2016); (iii) “Fully integrated” activation across the modalities, with somatotopy-specific brain responses in the motor cortex and overlapping brain responses in STG, possibly also with topographical organization (Pulvermüller and Fadiga 2010; Strijkers and Costa 2016; Pulvermüller 2018).

## Materials and methods

We have made the code, analyses pipelines, materials, averaged data and all statistical analyses of the processed data available in the project Open Science Framework (OSF) repository at *https://osf.io/bhfdw/* (the raw, preprocessed and structural MRI data, which are too large in size for the OSF repository, will be made available upon request to the corresponding author).

### Participants

Forty-four native French speakers with normal hearing, normal or corrected eyesight, and no history of any neurological problems participated in the study. We excluded 7 participants from the study due to technical issues, resulting in a total of 37 participants (female = 27, median age = 25.5, range = 18.5 to 36.2). All of the participants were recruited on a voluntary basis through the Aix-Marseille University mailing lists. The study received ethical approval (filed under Id 2017-A03614-49 from the regional ethical committee, Comité de protection des personnes sud Méditerranée I). All participants gave written consent to participate in the study and received monetary compensation for their participation.

### Stimuli

Twenty minimal phonological pairs of nouns were selected from the Lexique database on French language (New et al. 2004). Selection criteria included that minimal word pairs could only differ in their starting consonant, namely, bilabial “/b/ or/p/” for one member of the minimal pair, and an alveolar consonant, “/d/ or/t/”, for the other member. Items were controlled for length (1 to 3 syllables), lexical frequency, semantic category (including the absence of any stimuli with sound-related meaning given that the focus of our study was phonology; see OSF: https://osf.io/bhfdw/), and had to be concrete, depictable nouns (since the same stimuli were presented in auditory and visual modality; see below). Analyses of the naming latencies in the production task (standard picture naming) furthermore confirmed that the items were indeed well-controlled since no differences in reaction times were found between the bilabial words (783 ms) and alveolar words (785 ms) (details as well as all individual naming latencies for all items can be found in the online repository: https://osf.io/bhfdw/)*.* An additional set of 10 filler nouns and 5 practice nouns were selected which, unlike the target pairs, did not start with alveolar or bilabial consonants. All stimuli were presented in the visual modality for the production task (object naming) and the auditory modality for the perception task (passive listening) (see https://osf.io/bhfdw/ for an overview of all stimuli).

For the visual modality, 40 gray-scale PNG images depicting the minimal pairs (targets), as well as 10 filler images, and 5 practice images were selected. Out of those, 37 images (22 targets, 10 filler items, and 5 practice items) were selected from the MultiPic database (Duñabeitia et al. 2018), and an additional 18 target images were selected from the open-source gray-scale images database Google Line Drawings. All images were matched in pixel-size.

For the auditory modality, the target, filler and practice stimuli were pre-recorded by a native female French speaker in the soundproof room of the Laboratoire Parole et Langage at Aix-Marseille University using an RME fireface UC audio interface and a headset Cardioid Condenser Microphone (AKG C520) at a sampling rate of 44,100 Hz. The tokens were preprocessed and denoised via AudaCity® software. Average duration of audio stimuli was 640 ms (range: 304 to 841 ms), and there were no differences in average length between the bilabial and alveolar stimuli sets.

Prior to the experiment (and outside the scanner) the participants were familiarized with all target and filler items by presenting randomly each object accompanied with its corresponding auditory name. This was done to substantially reduce potential naming errors and potential repetition effects during the main experiment (since errors and repetition effects are drastically reduced for objects and words after the first repetition; eg Strijkers et al. 2010). During the main experiment, each target and filler appeared eight times for each participant (four times in the production task and four times in the perception task), leading to a total of 400 trials (160 target trials +40 filler trials in production and 160 target trials +40 filler trials in perception). In this manner, for each task, there were four runs (40 targets +10 fillers), which were semi-randomized for each run following the constraint that the same starting consonant (/b/,/p/,/d/and/t/) could not be presented in sequence for more than two times.

### Tasks and procedure

The main experiment consisted of two tasks, a standard production task and a standard perception task (Fig. 1).

“The production task” was object naming, where participants are presented with an object picture for which they have to utter its most typical designation or “name” as rapidly and correctly as possible. Trial structure was the following (Fig. 1, “upper panel”): A fixation cross was presented in the center with variable duration between trials (randomly varying between 500 and 1,200 ms to create inter-stimulus jitter). This was followed by object presentation for 640 ms (which corresponds to the average duration of the auditory stimuli in the perception task to ensure that overall target duration was the same between the production and perception tasks). Object presentation was followed by a blank screen presented for 2,500 ms.

“The perception task” was a go/no-go paradigm where the no-go trials corresponded to the auditory presentation of the target stimuli (passive listening; 80% of the trials), and the go-trials included the auditory presentation of the filler stimuli followed by two alternative pictures (20% of the trials) (Fig. 1, “lower panel”). On those go-trials participants had to perform word-to-picture matching to ensure semantic processing of the auditory input: the two images following the filler word appeared on the left and right of the screen and participants had to push either a left or right response button to indicate which object corresponded to the just heard word. Participants had to press the response buttons with their left middle and index fingers. All images were chosen from the pool of filler stimuli, and response fingers were fully counterbalanced across participants. For analyses, only the no-go trials (passive listening) are analyzed. Trial structure of the no-go trials fully matched that of the production task (Fig. 1): A trial started with jittered fixation cross presentation (between 500 and 1,200 ms), followed by the audio stimulus, which was presented on average for 640 ms (range: 304 to 841 ms), and ending with a blank screen during 2,500 ms. In the case of the filler trials, instead of a blank screen after the audio stimulus the participants were presented with two images for 2,500 ms during which the word-to-picture decision could be made. For each run a baseline period of 5,040 ms at the beginning and 12,000 ms at the end was introduced.

### Functional localizer tasks

At the end of each session 2 functional localizer tasks using a blocked design were performed in counterbalanced order. One of the localizer tasks was designed to assess within each individual the spatial dissociation in the motor cortex between lip movements and tongue movements (based on the lip-tongue motor localizer in Pulvermüller et al. 2006). This was done to obtain functional ROIs for the bilabial (lip-related) vs. alveolar (tongue-related) conditions in the main experiment. Each of the two runs consisted of eight blocks of 16 s each; during one block the word “levres” (lips) was presented on the screen, while during the other block the word “langue” (tongue) was presented on the screen. Participants were instructed to slightly move their lips for the entire duration that the word “levres” was presented, and slightly tap their tongue on the palate for the entire duration the word “langue” was presented. No breaks were introduced between the blocks, and baselines of 5,040 ms and 13,040 ms were recorded at the beginning and end of each run respectively.

In the other localizer task participants listened to syllables, which either started with a bilabial or alveolar speech sound to define individual specific functional ROIs underlying the phonological processing of bilabial and alveolar consonants in the superior temporal gyrus. Each of the two runs consisted of 8 blocks of bilabial-initial “(/pa/,/ba/,/ma/)” or alveolar-initial “(/da/,/ta/,/sa/,/za/,/na/)” syllables, and each block lasted for 16 s.

### MRI data acquisition

Functional images were acquired at the Marseille MRI Center, using a 3-T Siemens Magnetom Prisma MR system with a 64-channel head coil. Functional images covering the whole brain were acquired during task performance, using a multiband blood oxygen level dependent (BOLD)-sensitive gradient echo planar imaging (EPI) sequence (Moeller et al. 2010) (repetition time [TR] = 1,040 ms, echo time [TE] = 35.2 ms, flip angle = 62°, 65 slices, field of view [FOV] = 200 × 200 mm2, matrix = 100 × 100, slice thickness = 2 mm, multiband factor = 5, bandwidth = 2,380 Hz/pixel). Whole-brain anatomical MRI data were acquired using high-resolution structural T1-weighted (T1w) image (MPRAGE sequence, voxel size = 1 × 1 × 1 mm3, data matrix 256 × 240 × 192, TR/TI (inversion time)/TE = 2,300/925/2.98 ms, flip angle = 9°, bandwidth = 240 Hz/pixel, GRAPPA = 3). Prior to functional imaging and to correct functional images for susceptibility induced distortions, a pair of spin-echo EPI sequences was acquired twice with opposite phase encode directions along the anterior–posterior axis with the following parameters: TR/TE = 7,106/58 ms, voxel size = 2 × 2 × 2 mm3, slices = 65, FOV = 200 × 200 mm2. Participants’ movements during data acquisition were controlled using Framewise Integrated Real-time MRI Monitoring (Dosenbach et al. 2017).

### Image processing and analyses

MRI data were preprocessing performed using *fMRIPrep* 20.2.2 (RRID:SCR_016216) (Esteban et al. 2019), which is based on “Nipype” 1.6.1 (RRID:SCR_002502) (Gorgolewski et al. 2011).

### Anatomical data preprocessing

The T1w image was corrected for intensity non-uniformity with N4BiasFieldCorrection (Tustison et al. 2010), distributed with antsApplyTransforms (ANTs) 2.3.3 (RRID:SCR_004757) (Avants et al. 2009), and used as T1w-reference throughout the workflow. The T1w-reference was then skull-stripped with a “Nipype” implementation of the antsBrainExtraction.sh workflow (from ANTs), using OASIS30ANTs as target template. Brain tissue segmentation of cerebrospinal fluid (CSF), white-matter (WM) and gray-matter (GM) was performed on the brain-extracted T1w using fast (FSL 5.0.9, RRID:SCR_002823). Volume-based spatial normalization to one standard space (MNI152NLin2009cAsym) was performed through nonlinear registration with antsRegistration (ANTs 2.3.3), using brain-extracted versions of both T1w reference and the T1w template. The following template was selected for spatial normalization: “ICBM 152 Nonlinear Asymmetrical template version 2009c” (RRID:SCR_008796; TemplateFlow ID: MNI152NLin2009cAsym).

### Functional data preprocessing

For each of the 16 BOLD runs found per subject (2 experimental tasks and 2 localizers), the following preprocessing was performed. First, a reference volume and its skull-stripped version were generated by aligning and averaging 1 single-band references. Head-motion parameters with respect to the BOLD reference (transformation matrices, and six corresponding rotation and translation parameters) are estimated before any spatiotemporal filtering using mcflirt (FSL 5.0.9). A B0-nonuniformity map (or “fieldmap”) was estimated based on two (or more) EPI references with opposing phase-encoding directions, with 3dQwarp Cox and Hyde (AFNI 20160207). Based on the estimated susceptibility distortion, a corrected EPI reference was calculated for a more accurate co-registration with the anatomical reference. The BOLD time-series (including slice-timing correction when applied) were resampled onto their original, native space by applying a single, composite transform to correct for head-motion and susceptibility distortions. These resampled BOLD time-series will be referred to as “preprocessed BOLD in original space”, or just “preprocessed BOLD”. The BOLD reference was then co-registered to the T1w reference using flirt (FSL 5.0.9) with the boundary-based registration cost-function. Co-registration was configured with nine degrees of freedom to account for distortions remaining in the BOLD reference. First, a reference volume and its skull-stripped version were generated using a custom methodology of “fMRIPrep”. Several confounding time-series were calculated based on the “preprocessed BOLD”: framewise displacement (FD), differential variance (DVARS) and three region-wise global signals. FD was computed using two formulations following Power (absolute sum of relative motions) (Power et al. 2014) and Jenkinson (relative root mean square displacement between affines) (Jenkinson et al. 2002). FD and DVARS are calculated for each functional run, both using their implementations in “Nipype”. The three global signals are extracted within the CSF, the WM, and the whole-brain masks. Additionally, a set of physiological regressors were extracted to allow for component-based noise correction (“CompCor”) (Behzadi et al. 2007). Principal components are estimated after high-pass filtering the “preprocessed BOLD” time-series (using a discrete cosine filter with 128 s cut-off) for the two CompCor variants: temporal (tCompCor) and anatomical (aCompCor). tCompCor components are then calculated from the top 2% variable voxels within the brain mask. For aCompCor, three probabilistic masks (CSF, WM, and combined CSF + WM) are generated in anatomical space. The implementation differs from that of Behzadi et al. in that instead of eroding the masks by 2 pixels on BOLD space, the aCompCor masks are subtracted a mask of pixels that likely contain a volume fraction of GM. This mask is obtained by thresholding the corresponding partial volume map at 0.05, and it ensures components are not extracted from voxels containing a minimal fraction of GM. Finally, these masks are resampled into BOLD space and binarized by thresholding at 0.99 (as in the original implementation). Components are also calculated separately within the WM and CSF masks. For each CompCor decomposition, the *k* components with the largest singular values are retained, such that the retained components’ time series are sufficient to explain 50% of variance across the nuisance mask (CSF, WM, combined, or temporal). The remaining components are dropped from consideration. The head-motion estimates calculated in the correction step were also placed within the corresponding confounds file. The confound time series derived from head motion estimates and global signals were expanded with the inclusion of temporal derivatives and quadratic terms for each. Frames that exceeded a threshold of 0.5 mm FD or 2.0 standardized DVARS were annotated as motion outliers. The BOLD time-series were resampled into standard space, generating a “preprocessed BOLD run in MNI152NLin2009cAsym space”. First, a reference volume and its skull-stripped version were generated using a custom methodology of “fMRIPrep”. All resamplings can be performed with “a single interpolation step” by composing all the pertinent transformations (ie head-motion transform matrices, susceptibility distortion correction when available, and co-registrations to anatomical and output spaces). Gridded (volumetric) resamplings were performed using ANTs, configured with Lanczos interpolation to minimize the smoothing effects of other kernels. Non-gridded (surface) resamplings were performed using mri_vol2surf (FreeSurfer). Many internal operations of “fMRIPrep” use “Nilearn” 0.6.2 (RRID:SCR_001362) (Abraham et al. 2014), mostly within the functional processing workflow.

### Analyses

The preprocessed data were imported to and analyzed using the Statistical Parametric Mapping software (SPM12; https://www.Fil.ion.ucl.ac.uk/spm/software/spm12/) in MATLAB R2018b (Mathworks Inc., Natick, MA). Smoothing was performed with an isotropic Gaussian kernel (full-width at half-maximum = 5 mm). For the univariate ROIs analyses a General Linear Model (GLM) was built for each participant for each experimental task (production and perception), as well as for the motor and syllable localizers tasks. Regressors of no interest included 24 head movement regressors, the mean signal of the CSF and WM mask, and 24 aCompCor regressors related to CSF and WM.

**Fig. 2:**
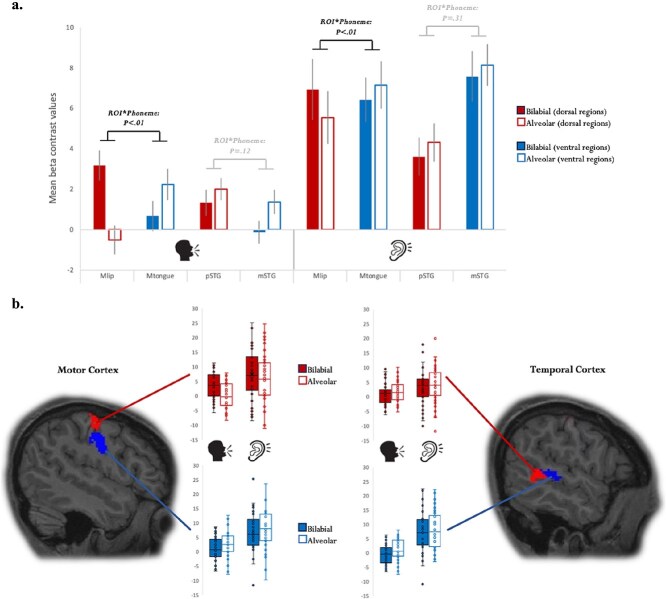
Results of the phoneme contrast between production and comprehension. a) Mean voxel activation (with mean standard error) for bilabial-initial words (filled red and blue bars) and alveolar-initial words (outlined red and blue bars) in production (left) vs. comprehension (right) for the different ROIs in the motor cortex (Mlip = lip-region in motor cortex; Mtongue = tongue-region in motor cortex) and temporal cortex (pSTG = posterior superior temporal gyrus; mSTG = middle superior temporal gyrus). The graph highlights the interactions between ROI and the phoneme conditions which are identical across the language modalities: significant interactions in the motor cortex and the absence of significant interactions in the temporal cortex. b) Dispersion graphs of the mean voxel activity of each participant for the different phoneme conditions (bilabial vs. alveolar) in each of the four ROIs (motor regions left, temporal regions right) in production and comprehension. The graphs visualize the remarkable overlap between production and comprehension for the bilabial-alveolar contrast in all four ROIs. In the motor cortex, the red mask corresponds to the lip-region and the blue mask to the tongue-region. In the temporal cortex, the red mask corresponds to a more posterior portion of the superior temporal cortex and the blue mask to a more middle portion of the superior temporal cortex (note that the ROIs are based on individual masks).

For the motor localizer task, the GLM included, for each of the 4 runs, the following regressors of interest: “lip-movement” and “tongue-movement”. To calculate the functional ROIs corresponding to the lips and the tongue within the motor cortex, individual maps were extracted based on the GLM output of the direct contrasts (contrasts: “lip-movement” vs. “tongue-movement”; and “tongue-movement” vs. “lip-movement”). In addition, to avoid including nonmotor related activity, we added an anatomical constraint to only consider activity within the sensorimotor cortex (based on the 400-parcels atlas of Schaefer et al. 2018). In this manner, our functional ROIs concerned masks of the intersection between functional and anatomical constraints within each individual (Fig. 2).

For the auditory syllable localizers, the GLM did not result in any reliable bilabial vs. alveolar ROIs within the temporal cortex. Note that for the localizers we tried both the standard individual functional ROIs approach as well as the group-constrained subject-specific approach developed by Fedorenko et al. (2010). While for the motor localizer results were comparable, for the auditory localizer neither approach produced significant dissociable ROIs in the temporal cortex. Even when we lowered our threshold from *P* < 0.001 to *P* < 0.05 (uncorrected) in order to see whether a more lenient approach would uncover functional ROIs for the bilabial vs. alveolar sound contrast still no significant ROI could be identified. This indicates that our auditory syllable task is likely not a good localizer task to highlight differences in phoneme processing within the temporal cortex and/or that place of articulation cannot be localized in a topographical manner in the superior temporal cortex (eg Mesgarani et al. 2014; Leonard et al. 2024; see also the General Discussion). Therefore, we defined the ROIs in the temporal cortex based on the intersection of the individual GM with predefined anatomical regions from the 400-parcel atlas of Schaefer et al. (2018). Concretely, based on the previous literature (Pulvermüller et al. 2006; Strijkers et al. 2017) we selected two anatomical parcels in the temporal cortex corresponding to the middle portion of the STG (parcel 197) and the posterior portion of the STG (parcel 198).

For the ROI analyses of the main experiment, GLMs were created for each of the 8 runs and each of the 4 ROIs identified above, which contained the following regressors of interest: “bilabial_production”, “alveolar_production”, “bilabial_perception”, “alveolar_perception”, “filler_production”, and “filler_perception”. Within each of the four ROI masks we extracted for each of the regressors the mean voxel activation within each participant. For statistical analyses the contrast values of the experimental variables were created by subtracting the experimental regressors from the filler regressors (ie bilabial_production–filler_production; alveolar_production–filler_production; bilabial_perception–filler_perception; alveolar_perception–filler_perception). Analysis of variance (ANOVA) were run starting with all experimental variables, and subsequently breaking down those variables which displayed significant interactions. In this manner, the highest-level analysis concerned a 4-way 2x2x2x2 ANOVA including the following variables (with the corresponding levels in brackets): “Phoneme” (bilabial vs. alveolar) x “Area” (motor cortex vs. superior temporal cortex) x “ROI” (anterior [tongue-related] vs. posterior [lip-related]) x “Modality” (production vs. comprehension).

## Results

For clarity, here we will only report the results for the interactions of relevance for our hypotheses. The detailed results of all ANOVAs can be consulted in the online repository: https://osf.io/bhfdw/*.*

Starting with the 4-way ANOVA, which includes all variables tested in this study, namely “Phoneme” (bilabial vs. alveolar) x “Area” (motor cortex vs. superior temporal cortex) x “ROI” (anterior [tongue-related] vs. posterior [lip-related]) x “Modality” (production vs. comprehension), we observed significant 3-way interactions between “Phoneme”, “Area”, and “ROI” (F(1,36) = 35.53, MSE = 2.62, *P* < 0.001), and between “Phoneme”, “ROI”, and “Modality” (F(1,36) = 15.83, MSE = 3.54; *P* < 0.001). In subsequent 3-way ANOVAs split up by “Modality”, we observed significant 3-way interactions between “Phoneme”, “Area” and “ROI” in “production” (F(1, 36) = 25.79, MSE = 3.34; *P* < 0.001), and in “perception” (F(1,36) = 7.21, MSE = 2.66, *P* = 0.01)*.* Considering these significant interactions, next, and most crucially for the hypotheses, we performed 2-way ANOVAs split up by “Modality” and “Area” to assess whether the phoneme factor interacts with ROI across the language modalities and across the frontal and temporal cortices. In the “motor cortex” for “production” we observed a significant interaction between “Phoneme” and “ROI” (F(1,36) = 56.31, MSE = 4.34, *P* < 0.001), indexing stronger activation of the Lip Motor Cortex when naming objects starting with bilabial speech sounds and stronger activation of the Tongue Motor Cortex when naming objects starting with alveolar speech sounds (Fig. 2a and b). Importantly, also for “perception” the interaction between “Phoneme” and “ROI” was significant (F(1,36) = 9.06, MSE = 2.43, *P* = 0.005), likewise indicating that when participants passively listened to words starting with bilabial speech sounds there was more activity in the Lip Motor Cortex compared to words starting with alveolar speech sounds and vice versa in the Tongue Motor Cortex when listening to words starting with alveolar speech sounds (Fig. 2a and b). Finally, when running 2-way ANOVAs in the “temporal cortex” there was no significant interaction between “Phoneme” and “ROI” in “production” (F(1,36) = 2.59, MSE = 2.44, *P* = 0.12)*,* and no significant interaction between “Phoneme” and “ROI” in “perception” (F(1,36) = 1.07, MSE = 2.11, *P* = 0.31), indicating similar activation profiles for bilabial and alveolar speech sounds across the modalities in the STG (Fig. 2a and b); a result further confirmed by the absence of a significant interaction between “Phoneme” and “Modality” in the temporal cortex (F(1,36) < 1) (Fig. 2a). Instead, we observed a main effect of “Phoneme” in the temporal cortex (F(1,36) = 4.44, MSE = 7.52, *P* = 0.04) (Fig. 2a), with stronger activations for words starting with alveolar speech sounds compared to bilabial speech sounds in both modalities and both regions of the STG (Fig. 2b).

## Discussion

In this fMRI study we set out to test whether the production and comprehension of words recruit the same phonology network across the language modalities. Thirty-seven participants performed both object naming and passive listening tasks on the same word stimuli. To trace cortical regions sensitive to phonological processing we used minimal pairs, which solely differed in their initial phoneme: bilabial (eg “ballon”) or alveolar (eg “talon”). When comparing this phoneme contrast between the production and comprehension tasks, similar region by phoneme interactions were found in the motor cortex and the superior temporal cortex. In motor cortex a phoneme-specific topographical response was found for language production with bilabial words eliciting stronger activation in the motor region sensitive to lip movements and alveolar words eliciting stronger activation in the motor region sensitive to tongue movements (Fig. 2). Crucially, the phoneme by motor region interaction was also significant in the passive listening task, showing that within the same individuals those motor regions that responded in a phoneme-specific topographical manner during production, also responded in a phoneme-specific topographical manner during comprehension (Fig. 2). Furthermore, also in the superior temporal cortex we observed a similar activation pattern for the phoneme manipulation during object naming and passive listening: both in more posterior as more middle portions of the STG alveolar-initial words produced stronger activations compared to bilabial-initial words (Fig. 2). Our study demonstrates a phoneme-specific fronto-temporal network when producing words which overlaps with the network elicited when listening to words. This data pattern supports brain language models, which assume that the phonological processing of words is integrated across the language modalities (Pulvermüller and Fadiga 2010; Strijkers and Costa 2016; Pulvermüller 2018) (Fig. 1).

According to such integration models word representations will rely (at least) on temporal and frontal regions both when uttering as when listening to language. This is because this class of models are based on assembly coding, which allows to integrate correlated sensory–motor activation into neuronal circuits whose neurons are widely distributed across sensory, motor and convergence areas and strongly interlinked by long-distance cortico-cortical connections (Hebb 1949; Braitenberg 1978; Artola and Singer 1993). When extending this neurobiological principle to cognition and language (Pulvermüller 1999), co-processed auditory and motor properties of word forms become critical for interlinking perception- and production-related neurons in auditory and motor systems due to consistent correlated activation during acquisition (Werker and Tees 1999; Kuhl 2010). Therefore, in processing words, phonological information about the speech sounds making up the word form are processed in parallel in auditory brain regions in temporal cortex (acoustic-phonological features) and in motor-related brain regions in fronto-central cortex (articulatory-phonological features; Pulvermüller and Fadiga 2010). In this manner, the phoneme networks that sustain word representations are fully shared between language production and comprehension (above and beyond the fact that the starting point of comprehension is acoustic and the end point of production is articulation). Recent evidence supports such parallel fronto-temporal phonological processing in speech production (Strijkers and Costa 2016; Kerr et al. 2023) and comprehension (Pulvermüller 2018), along with its emergence in the first year of life (Kuhl 2010). The results of the current study align with such integrative view, as they assume a shared neural space underpinning both word production and perception.

Observing somatotopic motor activations when listening to speech replicates previous fMRI studies (Wilson et al. 2004; Skipper et al. 2007; Correia et al. 2015; Evans and Davis 2015; for a review see Schomers and Pulvermüller 2016), but also extend those studies in important ways. Firstly, whereas previous neuroimaging work focused on the perception of meaningless syllables, we here demonstrate phoneme-specific motor activity elicited by meaningful words. Considering that words come with much greater phonetic variability compared to repeated syllables, observing phoneme-specific dissociations in motor cortex in the current set-up demonstrates robustness of the findings. It also addresses speculations that motor cortex involvement in speech comprehension might only be observable in artificial phonetic tasks, such as sound detection or classification, which are indeed not most relevant in day-to-day conversation (Mahon and Caramazza 2008; Hickok 2014a). In contrast, producing or listening to meaningful language, as is the case here, concerns a more natural way of processing phonemes. A further noticeable extension of the current study compared to previous work is that both the comprehension and production of the same words were explored within the same brains and in an individual-specific manner. This leaves little doubt that the same motor regions recruited for the speech planning of the alveolar and bilabial minimal pairs are also recruited when passively listening to these words. Taken together, our study provides compelling support for the motor cortex’s involvement in language comprehension.

Turning to the superior temporal cortex, both in posterior and middle portions of STG alveolar-initial words produced greater activation compared to bilabial-initial words during word production and comprehension. While, crucially, this activation pattern for phonological processing was again the same in production and comprehension, the pattern was different from the one found in the motor cortex in that the activation was not phoneme-specific. In the introduction, we had suggested that similar region by phoneme interactions predicted for the motor cortex may also be present in the temporal cortex. This suggestion was based on (post hoc) observations from previous work (Pulvermüller et al. 2006; Strijkers et al. 2017), and the theoretical consideration that if phonological networks include sensory–motor integration across production and perception, the spatial dissociations between alveolar and bilabial speech sounds in motor cortex may be communicated to temporal cortex in a topographic manner due to the general neighborhood-preserving nature of cortico-cortical connections (Braitenberg and Schuz 1998). Categorical responses to different phonemic properties in the superior temporal cortex are well established (Obleser et al. 2003; Chang et al. 2010; Arsenault and Buchsbaum 2015), though not necessarily for place of articulation, at least when considering comprehension (Bhaya-Grossman and Chang 2022). The absence of a dissociable superior-temporal brain response for the alveolar—bilabial contrast in the present study could be due to other acoustic features than place of articulation being more dominant for the organization of phonemes in the superior temporal cortex (Mesgarani et al. 2014; Arsenault and Buchsbaum 2015; Cheung et al. 2016; Leonard et al. 2024).

Despite the absence of topographic specificity of the brain response to the alveolar and bilabial stop consonants in superior temporal cortex, the activation again revealed the same pattern in language production and comprehension, with more activation for alveolar-initial words compared to bilabial-initial words across the modalities. Stronger neural responses to alveolar than bilabial speech sounds had previously been found in other production and perception studies (Pulvermüller et al. 2006; D'Ausilio et al. 2009;Bartoli et al. 2015 ; Strijkers et al. 2017). This effect could be related to alveolar speech sounds having overall greater variability than bilabial speech sounds (Chomsky and Halle 1968), and alveolar speech sounds being produced by a biomechanically more complex articulator (tongue) compared to bilabial speech sounds (lips) (Bartoli et al. 2015; Strijkers et al. 2017). Regardless of the exact reason why alveolar speech sounds activated STG more strongly than bilabial speech sounds, the global picture that emerges from our work is that the shared phonological network of words across the modalities is subserved by more general distributed representations in the superior temporal cortex linked to more phoneme-specific local topographies in motor cortex. In a sense, this corresponds to a combination of panels (b) and (c) of the possible outcomes depicted in Fig. 1 where STG displays a distributed pattern across modalities and motor cortex a topographical pattern across modalities. And while we do not deny that for certain tasks or other acoustic features the motor cortex may also show distributed sensitivity (Cheung et al. 2016), at least when directly contrasting place of articulation for meaningful words as in this study, phoneme-specific topographical responses in the motor cortex are coupled with distributed responses in STG both when speaking and listening.

Integrating this overlapping spatial network observed for phonological processing with the fast parallel time-course of word processing observed across modalities (Fairs et al. 2021), suggests that words rely on the same neural organization and activation dynamics during both language production and comprehension. And while our data do not address the causality of the observed activations (but see eg Fadiga et al. 2002; D'Ausilio et al. 2009; Schomers et al. 2015), the overlapping temporal dynamics together with the overlapping spatial dynamics as observed in this study suggest at least that motor regions contribute to the perceptions of words and sensory regions contribute to the production of words. A common neural basis for words across the language modalities challenges traditional accounts and implies alternative mechanisms for differentiating production and comprehension in both healthy and brain-damaged individuals. One such mechanism could be that differences between production and comprehension arise not from distinct neural representations but from behavior-specific processing dynamics after word activation (Strijkers and Costa 2016; Fairs et al. 2021; Pickering and Strijkers 2024). The memory representation of a word would activate similarly during speaking and listening, but the subsequent goal-directed processing, such as articulating the word or integrating it in the prior context, would vary by language behavior. This framework, which combines shared memory representations across the language modalities with distinct goal-directed processing, may offer novel perspectives to explain important production-perception phenomena in the cognitive and neurosciences such as neuropsychological double dissociations between production and comprehension (eg Shallice 1988), how words are integrated across the modalities to achieve combinatorial processing (Hagoort and Indefrey 2014), how to engage in dialog (Pickering and Garrod 2021), and how we can monitor our speech for errors (Runnqvist et al. 2021; Runnqvist 2023).

## Conclusion

In this fMRI study with 37 participants, we asked whether phoneme-specific networks can be observed in both language production and comprehension. We found for both language modalities the same pattern of haeamodynamic brain activity specific to different phoneme types: minimal pairs differing only in the word-initial alveolar or bilabial phoneme activated the motor cortex topographically and the superior temporal cortex to different degrees in a distributed manner, regardless of whether participants were uttering or listening to those minimal pairs. We argue that this pattern meets the predictions of integrative neurobiological models of language (Pulvermüller 1999, 2018; Pulvermüller and Fadiga 2010; Strijkers 2016; Strijkers and Costa 2016; Kerr et al. 2023), according to which the cortical representations of words are neuronal circuits that are shared between language production and comprehension. The results indicate that language representations are not constrained by a strict sensory–motor division. Instead, our data suggest that language comprehension includes motor areas of the brain, and language production includes perceptual areas of the brain. And while phonology in the brain clearly encompasses more than the prediction-specific regions focused on here, our findings show that within the same individual phoneme-specific motor topography during speaking is also active during listening, and more distributed phoneme sensitivity in superior temporal cortex during listening manifests also when speaking. This study is a compelling test of the notion that word representations, including their phonological properties, are integrated across and general to both language modalities.
